# Serial ctDNA dynamics predict clinical outcomes in metastatic and locally advanced PDAC: a systematic review

**DOI:** 10.3389/fonc.2026.1901721

**Published:** 2026-07-30

**Authors:** Supriya Peshin, Ehab Takrori, Ibrahim Halil Sahin, Harrison Kim, Mark Rubinstein, Claire Verschragen, Zahra Hamedi, Shafia Rahman

**Affiliations:** 1 Department of Internal Medicine, Norton Community Hospital, Norton, VA, United States; 2 College of Medicine, Alfaisal University, Riyadh, Saudi Arabia; 3 Department of Gastrointestinal Medical Oncology, University of Michigan, Ann Arbor, MI, United States; 4 Department of Radiology, The Ohio State University, Columbus, OH, United States; 5 Pelotonia Institute for Immuno-Oncology, The Ohio State University Comprehensive Cancer Center-James Cancer Center and Solove Research Institute, Columbus, OH, United States; 6 Division of Medical Oncology, The Ohio State University Comprehensive Cancer Center, Columbus, OH, United States; 7 Division of Hematology and Oncology, Department of Medicine, University of California Irvine, Orange, CA, United States

**Keywords:** CA19-9, circulating tumor DNA, early molecular response, liquid biopsy, longitudinal monitoring, minimal residual disease, overall survival, pancreatic ductal adenocarcinoma

## Abstract

**Background:**

Circulating tumor DNA (ctDNA) is being studied for prognosis and treatment monitoring in pancreatic ductal adenocarcinoma (PDAC). Given the limitations of CA19-9, including non-secretion and confounding by cholestasis, we assessed whether serial ctDNA dynamics predict progression-free survival (PFS) and overall survival (OS) in localized and advanced PDAC, and whether ctDNA adds information to CA19-9.

**Methods:**

We conducted a PRISMA-guided systematic review of PubMed, Translational Research, MDPI, and JAMA. Eligible studies included patients with PDAC who had ≥2 ctDNA measurements during perioperative care, treatment, or surveillance and reported associations with OS, PFS, DFS/RFS, recurrence, progression, EMR, molecular progression, or lead-time versus imaging/CA19-9. Two reviewers screened 119 records, of which 17 studies met inclusion criteria. Because of heterogeneity in assays, sampling schedules, and reporting, results were synthesized narratively.

**Results:**

Across studies, ctDNA detectability and clinical utility varied according to disease stage, assay type, and sampling window. In resected PDAC, a CLIA-validated ddPCR KRAS assay study reported preoperative ctDNA detection in 49% of patients (29/59). In the same cohort, ctDNA detected during follow-up predicted recurrence with 90% sensitivity (95% CI, 74%-98%) and 88% specificity (95% CI, 62%-98%) and preceded clinical or radiographic recurrence by a median of 84 days (IQR, 25-146). In advanced PDAC treated with palliative chemotherapy, longitudinal sampling every 4 weeks showed that ctDNA monitoring identified progression before radiology in 20 of 30 patients (67%) with baseline-detectable ctDNA, with a median lead time of 23 days (P = 0.01). In the same cohort, CA19–9 increases, when present, showed a shorter median lead time of 9.5 days (P = 0.03), and ctDNA detected progression in some patients without a corresponding CA19–9 increase during therapy. Across the included studies, baseline ctDNA positivity was generally associated with higher-risk disease, whereas early ctDNA decline or clearance during treatment and postoperative or longitudinal ctDNA negativity were associated with more favorable outcomes.

**Conclusion:**

Serial ctDNA dynamics in PDAC were associated with recurrence, progression, treatment response, and survival across perioperative and systemic therapy settings. ctDNA appears most consistent as a postoperative MRD marker and may complement CA19–9 and imaging during treatment monitoring. Prospective studies are needed to standardize sampling schedules, response thresholds, and ctDNA-guided clinical use.

**Systematic review registration:** https://www.crd.york.ac.uk/PROSPERO/view/CRD420261321972, identifier CRD420261321972.

## 1 Introduction

Pancreatic ductal adenocarcinoma (PDAC) is associated with poor survival, largely because many patients present with advanced disease and because recurrence is common even after curative-intent treatment. Following surgical resection, approximately two-thirds of patients develop recurrence, and reported 5-year survival is approximately 20–30% despite modern perioperative strategies (1–4).

Existing tools for risk stratification and treatment assessment are imperfect. Cross-sectional imaging remains central for staging and response evaluation, but progression may not be apparent until tumor burden has increased (5, 6). CA19–9 is widely used in PDAC, although some patients have non-elevated baseline levels and others may have confounding elevations, including from cholestasis (6). These limitations have led to interest in blood-based markers that may detect recurrence or treatment failure earlier than imaging or CA19–9 alone.

Circulating tumor DNA (ctDNA) is the tumor-derived component of cell-free DNA and has been studied in PDAC for prognosis, recurrence surveillance, and treatment monitoring (2–4). In resected disease, CLIA-validated KRAS ctDNA assays have been used to assess recurrence risk after surgery (2). Tumor-informed assays have also been evaluated for molecular residual disease (MRD), with perioperative ctDNA detection associated with patient outcomes (3). Tumor-agnostic next-generation sequencing (NGS) approaches suggest that ctDNA detection may retain prognostic value even when tumor-informed testing is not used (4).

Single-timepoint ctDNA detection provides only limited information about disease behavior. Serial testing may be more informative because it can show whether ctDNA clears, declines, persists, or rises during treatment or surveillance. In advanced PDAC, longitudinal ctDNA monitoring has been associated with clinical outcomes and has been reported to detect progression before radiologic imaging in some cohorts (5, 6). Quantitative ctDNA levels have also been associated with tumor burden and survival in metastatic PDAC (7).

Despite these findings, ctDNA studies in PDAC remain difficult to compare. Assays differ by platform, tumor-informed status, gene targets, analytical thresholds, and sampling schedules. Studies also vary in how they compare ctDNA with CA19–9 and imaging. These differences make it difficult to define when ctDNA should be measured and what degree of change should be considered clinically actionable.

We therefore conducted a PRISMA-guided systematic review of serial ctDNA dynamics in PDAC. We aimed to assess whether longitudinal ctDNA changes are associated with OS, PFS, DFS/RFS, recurrence, or progression; whether ctDNA can identify recurrence or progression before imaging; and how serial ctDNA compares with CA19–9 in localized and advanced disease.

## 2 Methodology

### 2.1 Protocol and registration

This systematic review was designed *a priori* and prospectively registered on PROSPERO (Registration ID: CRD420261321972) before study selection and data extraction commenced. No protocol amendments were made after registration.

### 2.2 Reporting guidelines

This systematic review was prepared in accordance with the Preferred Reporting Items for Systematic Reviews and Meta-Analysis (PRISMA) 2020 statement (8). Where applicable, we also followed PRISMA guidance for reporting search strategies, study selection, and synthesis methods, including presentation of the study selection process in a PRISMA flow diagram.

### 2.3 Eligibility criteria

We prespecified eligibility criteria using a PICO-TT-based approach focused on serial ctDNA dynamics in PDAC. We included peer-reviewed, full-text English-language studies enrolling adults (≥18 years) with PDAC across any stage (resectable, localized, borderline resectable, locally advanced, or metastatic) that performed plasma-based ctDNA testing using any platform (e.g., ddPCR/dPCR, targeted NGS, broad sequencing) and reported ≥2 ctDNA measurements per patient (e.g., clearance, conversion, rise/decline, early molecular response, or molecular progression) as defined by each study. Eligible studies reported associations between serial ctDNA findings and clinically meaningful outcomes, including OS, PFS, DFS/RFS, and/or monitoring endpoints such as concordance with imaging and lead-time to radiographic progression/recurrence (with CA19–9 and imaging comparisons extracted when available). We excluded preclinical studies, reviews, editorials, protocols, abstracts, case reports, studies limited to single-timepoint ctDNA testing, and studies where PDAC-specific results could not be separated from other tumor types. The PICO-TT criteria is summarized in **Table 1**.

**Table 1:** PICO-TT framework.

Component	Inclusion	Exclusion
Population	Adults (≥18 years) with confirmed PDAC (resectable/localized, borderline resectable, locally advanced, or metastatic).	Non-PDAC malignancies without separately extractable PDAC data; pediatric populations; non-human/preclinical studies.
Intervention	Plasma ctDNA assessment using any analytic platform (tumor-informed or tumor-agnostic, ddPCR/dPCR, targeted NGS, broad sequencing).	Tissue-only genomics (no plasma ctDNA); cfDNA quantity-only studies without tumor-specific alterations; germline-only testing; studies focused solely on assay development without clinical correlation.
Comparison	Not required; when available: CA19–9 kinetics and/or radiographic assessment (RECIST or equivalent).	Single-timepoint ctDNA studies; studies with ctDNA measured only once without longitudinal change; studies where serial change could not be extracted per patient or per group.
Outcome	OS, PFS, DFS/RFS, and/or monitoring endpoints (e.g., lead-time to radiographic progression/recurrence, response/progression concordance).	No clinically meaningful outcomes and no monitoring endpoints.
Timeframe	Serial testing with ≥2 ctDNA measurements per patient (perioperative, on-treatment, and/or surveillance).	
Trial Type	Prospective and retrospective cohort studies. Case-control studies. Randomized controlled trials. Cross-sectional studies. Multicenter observational studies.	Systematic reviews, meta-analysis, case series, observational studies, editorials, books, and grey literature.

### 2.4 Information sources

PubMed/MEDLINE was used as the primary bibliographic database to identify eligible peer-reviewed studies evaluating serial ctDNA dynamics in PDAC. In addition, targeted supplementary searches were conducted in selected journal and publisher platforms, including Translational Research, MDPI, and JAMA, to capture relevant articles from sources known to publish liquid biopsy and oncology biomarker studies. Reference lists of included articles were also manually screened to identify additional eligible studies not retrieved by the electronic searches. The final searches were conducted up to the most recent search date prior to analysis.

### 2.5 Search strategy

The search strategy was developed *a priori* to capture peer-reviewed studies evaluating ctDNA in PDAC with an emphasis on serial or longitudinal sampling and outcome associations. We combined controlled vocabulary and free-text keywords related to PDAC, ctDNA/liquid biopsy, and longitudinal assessment, including recurrence, progression, molecular residual disease, surveillance, and treatment response. The PubMed/MEDLINE search used MeSH and free-text terms, while supplementary journal and publisher platform searches used adapted keyword-based strategies according to the functionality of each platform. All retrieved records were imported into EndNote for deduplication before screening. Deduplication was performed using EndNote’s automated “Find Duplicates” function, which identifies duplicate references based on matching bibliographic fields, including title, author, year, and publication details where available. The automated duplicate search did not identify any duplicate records; therefore, no records were removed before title and abstract screening. The complete search strategies and information sources are provided in **Table 2**.

**Table 2:** Summary of search strategies used across information sources.

Information source	Source type	Search terms/strategy	Search method	Filters	Records retrieved
PubMed	Bibliographic database	(“Pancreatic Neoplasms”[Mesh] OR “pancreatic ductal adenocarcinoma” OR PDAC OR “pancreatic cancer” OR “pancreatic adenocarcinoma”) AND (“Circulating Tumor DNA” OR ctDNA OR “circulating tumour DNA” OR (“cell free DNA” OR ctDNA) OR “liquid biopsy”) AND (serial OR longitudinal OR dynamic OR kinetic OR monitor OR “minimal residual disease” OR MRD OR surveillance OR “treatment response” OR progression OR recurrence OR perioperative)	Advanced filtered search and MeSH	None	108
Translational Research	Journal platform	pancreatic ductal adenocarcinoma, PDAC, circulating tumor DNA, ctDNA	Advanced filtered search	None	5
MDPI	Publisher platform	pancreatic ductal adenocarcinoma, PDAC, circulating tumor DNA, ctDNA	Advanced filtered search	None	1
JAMA	Journal/publisher platform	pancreatic ductal adenocarcinoma, PDAC, circulating tumor DNA, ctDNA	Advanced filtered search	None	5

### 2.6 Study selection

**Figure 1:**
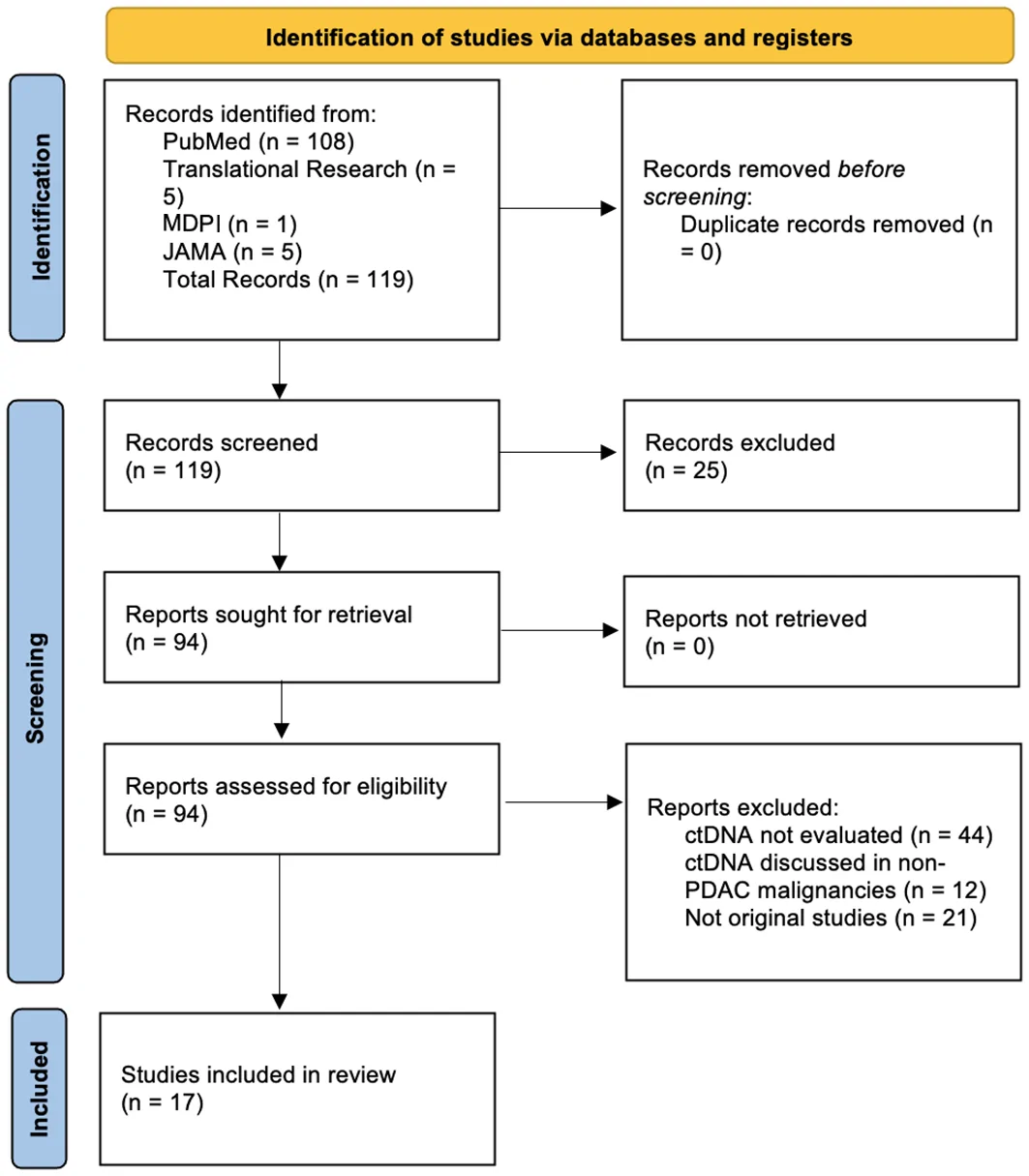
PRISMA flow diagram.

All records retrieved from PubMed/MEDLINE and supplementary journal/publisher platform searches were imported into EndNote and assessed for duplication using the automated “Find Duplicates” function. No duplicate records were identified. Two reviewers independently screened titles and abstracts against the prespecified eligibility criteria, followed by full-text assessment of potentially eligible articles. Disagreements at any stage were resolved through discussion and, when necessary, adjudication by a third reviewer. Reasons for exclusion at the full-text stage were documented. The overall selection process is summarized using a PRISMA 2020 flow diagram (**Figure 1**).

### 2.7 Data extraction

A standardized data extraction form was developed *a priori* and piloted on a subset of included studies. Two reviewers independently extracted data from each eligible article, and discrepancies were resolved by consensus. Extracted variables included: study characteristics (author, year, country, design, setting, sample size, inclusion period), patient characteristics (disease stage, treatment context, line of therapy, resection status), ctDNA methodology (platform and assay type, tumor-informed vs tumor-agnostic approach, gene targets such as KRAS/TP53, analytic thresholds/limits of detection, and ctDNA reporting metric [e.g., VAF, mutant allele fraction, copies/mL]), sampling strategy (timepoints, frequency, perioperative/on-treatment/surveillance windows), comparator assessments (CA19–9 kinetics, imaging schedule/RECIST when reported), and outcomes (OS, PFS, DFS/RFS, recurrence/progression detection and lead-time, response/progression classification, and multivariable-adjusted effect estimates including HRs/ORs with confidence intervals). When multiple publications reported overlapping cohorts, the most complete dataset was retained, and companion reports were used only to supplement missing details.

### 2.8 Outcomes

The primary outcomes were overall survival (OS) and progression-free survival (PFS) (or closely related time-to-event endpoints as reported by individual studies). Secondary outcomes included disease-free survival (DFS) and/or recurrence-free survival (RFS) in resected or curative-intent cohorts; ctDNA detectability at baseline and at prespecified clinical windows (perioperative, on-treatment, and surveillance); and longitudinal ctDNA monitoring endpoints, including ctDNA clearance, conversion (negative-to-positive or positive-to-negative), quantitative rise/decline (e.g., changes in variant allele fraction or mutant allele fraction), and study-defined early molecular response (EMR) or molecular progression. Additional secondary endpoints included lead-time of ctDNA-defined progression/recurrence relative to radiographic progression and/or CA19–9 kinetics, as well as concordance between ctDNA dynamics and radiographic response categories when available. Because ctDNA assays and response thresholds were heterogeneous, all ctDNA dynamics definitions were extracted and reported as defined by each study.

### 2.9 Risk of bias assessment

Risk of bias was assessed at the study level using the Quality In Prognosis Studies (QUIPS) tool, which is recommended for prognostic factor and prognostic biomarker reviews. Two reviewers independently evaluated each included study across the six QUIPS domains: study participation, study attrition, prognostic factor measurement (ctDNA assessment and timing), outcome measurement (OS/PFS/DFS/RFS and/or progression/recurrence determination), study confounding (e.g., stage, treatment regimen, performance status, tumor burden, CA19-9, and imaging schedule), and statistical analysis and reporting. Each domain was rated as low, moderate, or high risk of bias, and an overall risk-of-bias judgment was derived based on domain-level findings. Disagreements were resolved through discussion, with third-reviewer adjudication when necessary.

### 2.10 Data synthesis and statistical analysis

Given substantial heterogeneity across included studies, spanning ctDNA platforms (ddPCR/dPCR vs targeted or broad NGS), tumor-informed versus tumor-agnostic approaches, sampling schedules and clinical timepoints, PDAC stage and treatment context, and variable definitions of ctDNA response thresholds, we conducted a systematic review with narrative synthesis only, without meta-analysis. Before finalizing the synthesis approach, we assessed whether quantitative pooling was feasible for conceptually similar endpoints, including postoperative ctDNA positivity with DFS/RFS or OS, baseline ctDNA positivity with OS/PFS, and on-treatment ctDNA clearance or persistence with survival outcomes. Meta-analysis was considered appropriate only if studies were sufficiently comparable in clinical setting, ctDNA timepoint, assay definition, outcome definition, and reported effect measure. Specifically, pooling required studies to evaluate a comparable patient population, use a similar ctDNA assessment window, report a compatible endpoint, and provide extractable HRs or equivalent effect estimates with measures of uncertainty. These criteria were not met for the key endpoints because studies varied in perioperative versus surveillance timing, tumor-informed versus tumor-agnostic testing, PCR versus NGS platforms, positivity thresholds, survival endpoint definitions, and adjustment variables (2, 3, 5, 6, 11, 14, 15, 18). Studies were grouped *a priori* by clinical setting (resected/localized perioperative cohorts; non-metastatic cohorts receiving neoadjuvant therapy; locally advanced/metastatic cohorts receiving systemic therapy) and summarized according to the ctDNA dynamic feature evaluated (baseline detectability, clearance/conversion, quantitative rise/decline, study-defined early molecular response, molecular progression, and lead-time versus imaging and/or CA19-9).

Effect measures reported by individual studies (e.g., hazard ratios, odds ratios, median survival estimates, and lead-time metrics) were extracted and presented descriptively, with attention to adjustment variables and measurement windows. Because of this clinical and methodological heterogeneity, pooled random-effects estimates were not calculated. We also did not derive effect sizes from Kaplan-Meier curves, because reconstructed estimates would add imprecision and would not address the more fundamental incompatibility in ctDNA timing, assay thresholds, and endpoint definitions across studies.

For example, postoperative ctDNA positivity was evaluated across different clinical windows, including early postoperative MRD, post-adjuvant, and later surveillance timepoints, which may represent different biological states and recurrence risks. Similarly, advanced-disease studies assessed baseline detectability, quantitative ctDNA burden, early clearance, residual ctDNA, or molecular progression using different assays and treatment-monitoring schedules. Therefore, although several outcomes were conceptually similar, they were not sufficiently comparable for valid quantitative synthesis.

### 2.11 Assessment of reporting bias

Because this review used narrative synthesis without meta-analysis, formal statistical assessment of small-study effects and publication bias (e.g., funnel plots or Egger’s test) was not performed. Nevertheless, we qualitatively considered the potential for reporting bias by evaluating whether included studies selectively reported ctDNA endpoints, timepoints, or outcomes, and by comparing reported outcomes with study aims and methods when available. We also reviewed reference lists of included articles to identify additional eligible studies and reduce the likelihood of missing relevant evidence.

### 2.12 Certainty of evidence

Because of substantial clinical and methodological heterogeneity across studies, including differences in ctDNA platforms, gene targets, analytic thresholds, sampling schedules, and outcome definitions, and because this review used narrative synthesis without meta-analysis, a formal GRADE summary-of-findings assessment was not performed. However, to improve transparency, we conducted a structured qualitative certainty assessment across the major clinical use-cases. This assessment considered study design, QUIPS risk-of-bias judgments, consistency of findings across comparable clinical settings, directness of the ctDNA–outcome relationship, precision and completeness of effect reporting, and applicability to clinical practice (19–22).

Overall, the certainty of evidence was judged to be moderate for postoperative ctDNA positivity as a prognostic MRD marker in resected or localized PDAC. This conclusion was supported by consistent associations between postoperative ctDNA detection and recurrence or inferior survival across multiple cohorts, but certainty was limited by observational study designs, heterogeneous assays, variable postoperative sampling windows, and inconsistent adjustment for confounders (2, 3, 10, 14, 18).

The certainty of evidence was judged to be low-to-moderate for postoperative ctDNA negativity or clearance as a marker of lower recurrence risk, because findings were directionally consistent but were influenced by differences in assay sensitivity, timing of postoperative sampling, and the possibility of false-negative results in low-shedding tumors (2, 3, 10, 14, 18).

For locally advanced or metastatic PDAC, the certainty of evidence was judged to be low for on-treatment ctDNA dynamics as a monitoring biomarker. Although ctDNA decline, clearance, persistence, or rise was generally associated with treatment response, progression, PFS, or OS, studies varied in treatment setting, sampling interval, assay platform, molecular response definition, and comparator use with CA19–9 and imaging (5, 6, 11, 15, 16).

The certainty of evidence was judged to be low for ctDNA lead-time over imaging or CA19-9, because lead-time estimates are highly dependent on blood sampling frequency, imaging schedule, assay threshold, and whether CA19–9 was informative at baseline (2, 5, 6, 15).

Finally, the certainty of evidence was judged to be very low for ctDNA-guided treatment decision-making in PDAC. Current studies primarily establish prognostic and monitoring associations, while prospective interventional evidence demonstrating improved outcomes from ctDNA-triggered treatment escalation, de-escalation, or switching remains lacking (19, 20, 22).

## 3 Results

### 3.1 Study selection

The search of PubMed/MEDLINE and supplementary journal/publisher platform sources yielded 119 records (PubMed/MEDLINE n = 108, Translational Research n = 5, MDPI n = 1, JAMA n = 5). All records were imported into EndNote and assessed using the automated “Find Duplicates” function. No duplicate records were identified or removed; therefore, all 119 records proceeded to title and abstract screening. No duplicates were identified. After title, abstract, and full-text assessment against prespecified eligibility criteria, 77 records were excluded because ctDNA was not evaluated (n = 44), ctDNA was discussed in non-PDAC malignancies (n = 12), or the report was not an original study (n = 21). Ultimately, 17 studies met inclusion criteria and were included in the qualitative synthesis. The study selection process is summarized using a PRISMA 2020 flow diagram (**Figure 1**).

### 3.2 Study characteristics

The 17 included studies included prospective and retrospective cohorts across resected/localized, non-metastatic, locally advanced, and metastatic PDAC. Study contexts included perioperative or postoperative surveillance, neoadjuvant treatment, and systemic therapy monitoring. ctDNA assays included PCR-based approaches, most often targeting KRAS, as well as targeted or broader NGS platforms. Both tumor-informed and tumor-agnostic strategies were represented. Sampling schedules differed across studies and included perioperative testing, fixed-interval on-treatment monitoring, and surveillance after treatment. A concise summary of included studies is provided in **Table 3**. Detailed methodological characteristics, including full assay/platform descriptions, exact sampling timepoints, comparator assessments, and expanded outcome definitions, are provided in **Supplementary Table 1**. Key quantitative estimates, including reported HRs for survival endpoints, recurrence sensitivity/specificity, and lead-time estimates, are summarized narratively in the relevant Results and Discussion sections, while detailed study-level methodological and outcome information is provided in **Supplementary Table 1**.

**Table 3:** ​​Concise summary of included studies evaluating serial ctDNA dynamics in PDAC.

Study	Clinical setting	Assay approach	Serial sampling framework	Main clinical application
Huerta 2024	Metastatic PDAC	WES with ddPCR KRAS validation	Baseline and serial on-treatment monitoring	Treatment response monitoring; PFS/OS association; imaging and CA19–9 comparison
Mahadevia 2025	Advances PDAC	Plasma ctDNA NGS	Serial monitoring with tissue-plasma concordance assessment	Tissue-ctDNA concordance; response monitoring vs RECIST and CA19-9
Aaquist 2025	Resected PDAC	Tumor-informed ddPCR	Preoperative and postoperative surveillance sampling	Prognosis after surgery; RFS/OS association; ctDNA with or without CA19-9
Affolter 2021	Surgical PDAC	Tumor-uninformed NGS	Perioperative sampling	Prognostic value of perioperative ctDNA detection
Botta 2024	Resected stage I-III PDAC	Tumor-informed personalized mPCR-NGS	Diagnosis, postoperative MRD window, and surveillance	MRD and surveillance risk stratification; DFS prediction
Chen 2017	Unresectable stage III-IV PDAC	KRAS-enriched	Baseline and serial assessment during treatment	Treatment response monitoring; TTP/OS association; RECIST and CA19–9 comparison
Edland 2023	Advanced/inoperable PDAC	KRAS-focused qPCR	Baseline and fixed-interval chemotherapy monitoring	Earlier progression detection vs imaging; PFS/OS association
Groot 2019	Resected/localized PDAC	ddPCR KRAS	Perioperative and surveillance sampling	MRD detection; DFS and recurrence prediction
Jiang 2020	Surgical PDAC	Hybrid-capture NGS	Preoperative and postoperative surveillance sampling	MRD detection; DFS and recurrence prediction
Kim 2025	Metastatic PDAC	Targeted NGS	Baseline and response-evaluation sampling	Residual ctDNA as prognostic marker; PFS/OS association; CA19–9 comparison
Labiano 2024	Non-metastatic PDAC	cfDNA NGS with tumor profiling	Diagnosis, post-NAT assessment, and progression sampling	ctDNA dynamics during NAT; OS/EFS association
Lee 2019	Localized/resectable PDAC	SafeSeqS KRAS ctDNA assay	Preoperative and postoperative sampling	Postoperative recurrence risk; adjuvant chemotherapy benefit context
Maulat 2024	Resected PDAC	Tumor NGS with ddPCR KRAS	Portal/peripheral perioperative surveillance sampling	Compartment kinetics; recurrence and survival association
Murakami 2025	Resectable/borderline resectable PDAC	Targeted cfDNA NGS	Preoperative/perioperative sampling	Occult metastatic prediction; RFS/OS association
Perets 2018	Metastatic PDAC	Targeted KRAS sequencing	Serial follow-up sampling	Treatment monitoring; ctDNA dynamics vs CA19–9 and CT imaging
Strijker 2020	Treatment-naïve metastatic PDAC	Targeted NGS with ddPCR validation	Baseline with subset follow-up sampling	ctDNA quantity, tumor volume, and survival association
Sugimori 2020	Advanced PDAC	dPCR KRAS with selected NGS	Baseline and serial chemotherapy monitoring	Treatment monitoring; ctDNA dynamics vs RECIST and tumor markers

PDAC, pancreatic ductal adenocarcinoma; ctDNA, circulating tumor DNA; WES, whole-exome sequencing; ddPCR, droplet digital polymerase chain reaction; KRAS, Kirsten rat sarcoma viral oncogene homolog; PFS, progression-free survival; OS, overall survival; NGS, next-generation sequencing; RECIST, Response Evaluation Criteria in Solid Tumors; RFS, recurrence-free survival; mPCR-NGS, multiplex polymerase chain reaction next-generation sequencing; MRD, minimal residual disease; DFS, disease-free survival; PCR, polymerase chain reaction; TTP, time to progression; qPCR, quantitative polymerase chain reaction; NAT, neoadjuvant therapy; EFS, event-free survival; cfDNA, cell-free DNA; dPCR, digital polymerase chain reaction.

Detailed assay platforms, exact sampling timepoints, comparator assessments, and expanded definitions are provided in **Supplementary Table 1**.

### 3.3 Risk of bias assessment

Risk of bias was assessed using the QUIPS tool across six domains. Overall risk of bias was moderate in most studies, primarily due to cohort selection and incomplete control for confounding, as well as heterogeneity in ctDNA platforms and sampling schedules. Three studies were judged at high risk of bias because of small cohort sizes and limited confounder adjustment. Domain-level concerns most frequently involved study participation, confounding, and statistical analysis/reporting, whereas outcome measurement was generally well defined across studies. For studies primarily assessing ctDNA-tissue concordance, QUIPS ratings were applied to the extent that the study evaluated prognostic/monitoring outcomes; concordance-specific bias was interpreted qualitatively. All findings were recorded in **Table 4**.

**Table 4:** Risk of bias assessment of included studies using the QUIPS tool.

Study	Study participation	Study attrition	ctDNA measurement	Outcome measurement	Confounding	Analysis/reporting	Overall
Huerta 2024	Low	Moderate	Low	Low	Moderate	Moderate	Moderate
Mahadevia 2025	Moderate	Moderate	Low	Moderate	High	Moderate	Moderate
Aaquist 2025	Moderate	Moderate	Low	Low	Moderate	Moderate	Moderate
Affolter 2021	High	Moderate	Moderate	Low	High	High	High
Botta 2024	Moderate	Moderate	Moderate	Low	Moderate	Moderate	Moderate
Chen 2017	Moderate	Moderate	Moderate	Low	Moderate	Moderate	Moderate
Edland 2023	Moderate	Moderate	Moderate	Low	Moderate	Moderate	Moderate
Groot 2019	Moderate	Moderate	Low	Low	Moderate	Moderate	Moderate
Jiang 2020	High	Moderate	Moderate	Moderate	High	High	High
Kim 2025	Moderate	Moderate	Low	Low	Moderate	Moderate	Moderate
Labiano 2024	Moderate	Moderate	Moderate	Moderate	Moderate	Moderate	Moderate
Lee 2019	Moderate	Moderate	Low	Low	Moderate	Moderate	Moderate
Maulat 2024	Moderate	Moderate	Low	Moderate	Moderate	Moderate	Moderate
Murakami 2025	Moderate	Low	Moderate	Moderate	Moderate	Moderate	Moderate
Perets 2018	High	Moderate	Moderate	Low	High	High	High
Strijker 2020	Moderate	Moderate	Moderate	Low	Moderate	Moderate	Moderate
Sugimori 2020	Moderate	Moderate	Moderate	Low	Moderate	Moderate	Moderate

### 3.4 ctDNA assays and sampling frameworks

ctDNA testing methods varied across studies according to assay platform, tumor-informed status, and intended clinical use. PCR-based assays most often targeted recurrent KRAS alterations, while NGS-based approaches included targeted panels, broader sequencing, and commercial ctDNA assays. Both tumor-informed and tumor-agnostic strategies were represented (12, 13).

Tumor-informed approaches used tumor tissue to define patient-specific targets before plasma testing. These included personalized multiplex PCR-NGS assays, such as Signatera, reporting ctDNA as MTM/mL, as well as tumor sequencing followed by mutation-specific ddPCR at perioperative timepoints (3). Tumor-agnostic approaches used plasma-based sequencing without prior tumor-informed target selection, often incorporating error-suppression methods and, in some studies, matched leukocyte DNA to reduce false-positive findings.

Sampling schedules generally followed three patterns: perioperative or MRD testing before and after surgery; fixed-interval monitoring during systemic therapy, often alongside RECIST imaging and CA19-9; and pretreatment or neoadjuvant sampling for risk stratification (3, 5, 6, 9, 11). One perioperative study also compared portal and peripheral blood collected intraoperatively, followed by postoperative surveillance sampling (1).

Because assays, reporting units, and sampling intervals differed substantially, direct comparison across studies was limited. For this reason, results were synthesized narratively by clinical setting and intended use.

### 3.5 Baseline ctDNA detectability

Baseline ctDNA detection differed by disease stage and assay approach. Detection was generally less frequent in localized or perioperative PDAC cohorts than in advanced or metastatic disease, likely reflecting differences in tumor burden and ctDNA shedding (2, 5, 9, 14). Quantitative ctDNA measures, including VAF and MAF, were also associated with tumor volume and metastatic disease burden in studies that reported these comparisons (7).

Assay selection contributed to differences in baseline positivity. PCR-based assays, most commonly targeting KRAS, were used for sensitive mutation tracking, whereas NGS-based assays varied in gene content, error suppression, and reporting units (11, 15). In non-metastatic or resectable cohorts, baseline ctDNA status was often used as a starting point for evaluating later ctDNA clearance, persistence, postoperative MRD, or recurrence risk (10, 12, 13).

### 3.6 Perioperative ctDNA and MRD in resected/localized PDAC

In resected or localized PDAC, perioperative ctDNA was mainly studied as a marker of MRD and relapse risk. Most studies collected ctDNA at baseline or before surgery, again in early postoperative windows, and then during surveillance (1–3, 10, 14).

Postoperative ctDNA positivity was consistently associated with recurrence and worse RFS/DFS and OS. In contrast, postoperative negativity or clearance from detectable to undetectable ctDNA generally identified patients with lower recurrence risk (1–4, 10, 14). Baseline ctDNA positivity and persistence after surgery were also associated with earlier recurrence in several cohorts, suggesting that serial ctDNA may add risk information beyond a single preoperative result (2, 3, 10, 14).

Several studies assessed ctDNA together with CA19–9 and imaging, supporting its use as an additional surveillance marker rather than a replacement for standard follow-up (2, 3, 14). Most perioperative studies used peripheral blood, although one study also compared portal and peripheral blood collected intraoperatively to evaluate whether sampling closer to tumor drainage improved detection (1).

### 3.7 ctDNA dynamics during systemic therapy in locally advanced/metastatic PDAC

In locally advanced and metastatic PDAC, serial ctDNA was mainly studied during systemic therapy as a marker of response or progression. Most cohorts measured ctDNA at baseline and then at fixed intervals during chemotherapy, with changes reported as clearance, conversion to negativity, quantitative decline, persistence, or rise from nadir (5–7, 11, 15, 16).

Across studies, early ctDNA decline or clearance was associated with better outcomes, while persistent or rising ctDNA was associated with shorter PFS/OS and earlier progression (5, 6, 11, 15, 16). In several cohorts, ctDNA changes identified progression before radiographic confirmation, suggesting a possible lead-time advantage during treatment monitoring (5, 6, 11, 16).

Quantitative ctDNA measures also correlated with tumor burden. Higher baseline ctDNA levels, including VAF or MAF, were associated with larger disease burden and inferior survival in metastatic PDAC (7, 9). Although assays differed, including KRAS-targeted PCR methods and targeted NGS platforms, the overall pattern was similar: ctDNA clearance or decline favored better outcomes, while persistence or increase favored worse outcomes (5, 6, 9, 11, 15, 16).

### 3.8 Lead-time and concordance with imaging and CA19-9

Several studies compared serial ctDNA with imaging and CA19-9. In advanced or metastatic PDAC, ctDNA was often collected during chemotherapy and compared with RECIST-based imaging and tumor marker trends (5, 6, 11, 15, 17). ctDNA decline or clearance generally aligned with response, while persistent or rising ctDNA was associated with progression. In some cohorts, ctDNA suggested progression before radiographic confirmation, although reported lead time depended on imaging frequency and blood sampling intervals (5, 6, 11, 16).

In resected PDAC, postoperative ctDNA positivity was associated with later radiographic recurrence and was assessed alongside routine surveillance imaging (2, 3, 10, 14, 18). CA19–9 concordance varied across studies, supporting interpretation of ctDNA as an additional marker rather than a substitute for CA19–9 or imaging (5, 6, 10, 11, 15). Several studies also compared ctDNA findings with tissue profiling, supporting tumor-informed monitoring when tissue was available (1, 2, 12, 17). Differences in assays, reporting units, imaging schedules, and sampling frequency limited direct comparison of lead-time estimates across studies (3, 5, 7).

### 3.9 Special populations and subgroup findings

Subgroup analyses were reported in several settings. In resectable and borderline resectable PDAC, ctDNA was evaluated during neoadjuvant therapy and perioperative risk assessment, including as a marker of occult metastatic risk despite radiographically localized disease (10, 12, 13). Postoperative studies examined ctDNA by sampling window, including early MRD testing and later surveillance, and some assessed ctDNA clearance or persistence after surgery (2, 3, 14).

In advanced or metastatic PDAC, subgroup analyses focused on treatment regimen, baseline ctDNA burden, residual ctDNA at response assessment, tumor burden, and metastatic pattern (5, 7, 9, 15). One study also compared portal and peripheral blood sampling during surgery (1). Most subgroup findings were limited by small sample sizes and inconsistent definitions, so they should be considered exploratory.

### 3.10 Summary of key findings

Across 17 studies, serial ctDNA was associated with prognosis and disease monitoring in resected, locally advanced, and metastatic PDAC, although assay platforms, reporting units, and sampling schedules varied.

Baseline ctDNA detection was more common in advanced or metastatic disease than in localized disease and was associated with tumor burden in studies reporting quantitative measures (5, 7, 9). In resected PDAC, postoperative ctDNA positivity was associated with recurrence and worse survival, while postoperative negativity or clearance was associated with lower recurrence risk (1–3, 10, 14). During systemic therapy, ctDNA decline or clearance generally aligned with better outcomes, whereas persistent or rising ctDNA was associated with progression and shorter survival (5, 6, 11, 15, 16).

ctDNA was most useful when interpreted with imaging and CA19–9 rather than as a replacement for either. However, differences in assays, sampling timepoints, and confounder adjustment limit direct comparison across studies and support the need for prospective validation of clinically actionable thresholds (3, 7, 14).

## 4 Discussion

### 4.1 What this study adds

This review focuses on serial ctDNA change rather than single-timepoint detection in PDAC. Across the included studies, postoperative ctDNA was the most consistent setting in which ctDNA positivity was associated with recurrence and survival, supporting its role as an MRD marker in resected disease (2, 3). In advanced PDAC, ctDNA decline, clearance, persistence, or rise during treatment was associated with response and progression, but definitions and sampling schedules varied across studies (5). These findings support ctDNA as an additional marker to interpret alongside imaging and CA19-9, while also showing why prospective studies with standardized testing windows and response thresholds are needed. Importantly, conclusions should be interpreted by disease setting rather than generalized across all PDAC stages, because localized, perioperative, locally advanced, and metastatic cohorts differ in tumor burden, ctDNA shedding, treatment intent, and clinical decision points.

The main interpretive finding of this review is that serial ctDNA is not a single clinical tool but a biomarker strategy whose value depends on disease setting, assay context, and the intended clinical decision. In resected disease, ctDNA most directly addresses the problem of occult residual disease after apparently curative therapy, making postoperative MRD the most mature and clinically coherent use-case. In advanced disease, serial ctDNA more often reflects dynamic tumor burden and treatment pressure, making it useful for response assessment and early warning of progression, but less clearly actionable without prospective evidence that changing therapy based on molecular progression improves outcomes. Therefore, the clinical relevance of ctDNA should be judged not only by whether it correlates with survival, but by whether it can identify a decision point at which earlier intervention, closer surveillance, or trial enrollment could plausibly improve patient care (2, 3, 5, 15, 20).

### 4.2 Clinical meaning by use-case

#### 4.2.1 Perioperative MRD in localized/resected PDAC

In localized or resected PDAC, serial ctDNA is best interpreted as a marker of residual biological risk rather than simply as another surveillance test. The consistent association between postoperative ctDNA positivity and recurrence suggests that ctDNA may detect occult disease that is below the threshold of radiographic visibility. This is clinically important because resected PDAC remains characterized by high relapse risk despite curative-intent surgery and perioperative therapy. In this context, postoperative ctDNA positivity may help identify a subgroup with biologically persistent disease, while postoperative clearance or persistent negativity may identify patients with lower residual disease burden (2, 3, 10, 14, 18).

The major clinical question, however, is not whether postoperative ctDNA is prognostic, but how clinicians should act on a positive result. At present, the evidence supports risk stratification more strongly than treatment selection. A positive MRD result could justify closer surveillance, earlier imaging, or referral to ctDNA-directed trials, but routine escalation of adjuvant therapy based only on ctDNA remains investigational. Similarly, ctDNA negativity should not yet be used to de-escalate therapy outside a trial, because false-negative results may occur in low-shedding tumors or when sampling occurs outside the optimal postoperative window (3, 20).

Therefore, postoperative MRD represents the clearest near-term use-case for serial ctDNA in PDAC, but it also illustrates the central implementation gap. Current studies show that ctDNA can identify patients at higher risk of relapse, but they do not yet establish that ctDNA-guided intervention improves survival or quality of life. Future postoperative studies should move from prognostic validation toward interventional designs that test predefined ctDNA-triggered actions, such as intensified surveillance, adjuvant escalation, or clinical trial enrollment (19, 20, 22).

#### 4.2.2 On-treatment monitoring in locally advanced/metastatic PDAC

In locally advanced and metastatic PDAC, serial ctDNA appears to function less as an MRD marker and more as a dynamic measure of tumor burden, treatment sensitivity, and emerging progression. Across studies, ctDNA decline or clearance during systemic therapy generally tracked with better outcomes, whereas persistent or rising ctDNA suggested treatment resistance or increasing disease burden (5, 6, 11, 15, 16). This pattern is biologically plausible because ctDNA levels may change before anatomic progression becomes measurable, particularly when imaging intervals are long or radiographic response is difficult to interpret.

The clinical implication is that ctDNA may help identify patients whose disease biology is diverging from what is apparent on imaging or CA19-9. For example, rising ctDNA despite stable imaging may indicate molecular progression and could justify earlier repeat imaging, closer clinical reassessment, or trial consideration. Conversely, ctDNA decline during therapy may provide supportive evidence of treatment effect, particularly when imaging findings are equivocal. However, these interpretations should remain contextual rather than deterministic, because ctDNA shedding varies by tumor burden, metastatic pattern, assay sensitivity, and sampling interval (5, 7, 11, 15).

Importantly, on-treatment ctDNA monitoring in advanced PDAC is not yet equivalent to a validated treatment-switching strategy. A molecular rise may precede radiographic progression, but earlier detection does not automatically translate into improved outcomes unless an effective alternative intervention is available and tested prospectively. Therefore, ctDNA in advanced disease should currently be viewed as a complementary monitoring biomarker that may refine clinical suspicion and timing of reassessment, while ctDNA-directed therapy switching should be reserved for prospective trials or carefully defined investigational workflows (20).

#### 4.2.3 Lead-time and integration with CA19–9 and imaging

Lead-time should be interpreted carefully in ctDNA studies. Although several cohorts suggest that ctDNA may detect recurrence or progression before imaging, the magnitude of lead-time depends heavily on the frequency of blood sampling, the timing of scheduled imaging, the assay threshold used to define molecular progression, and whether CA19–9 was informative at baseline (2, 5, 6, 15). A longer molecular lead-time is clinically meaningful only if it creates an actionable window in which earlier imaging, treatment modification, or clinical trial enrollment can improve outcomes.

The most practical near-term role for ctDNA is therefore not replacement of CA19–9 or imaging, but refinement of existing monitoring pathways. Concordant worsening of ctDNA, CA19-9, and imaging increases confidence that progression is occurring. Discordant results are more challenging: rising ctDNA with stable imaging may warrant earlier repeat imaging or closer surveillance, whereas rising CA19–9 with negative ctDNA may reflect non-tumor factors such as biliary obstruction or may indicate disease not captured by the assay. Similarly, ctDNA negativity should not exclude recurrence when clinical or radiographic suspicion is high, particularly in localized disease where ctDNA shedding may be low (5, 6, 10, 15).

This synthesis supports a cautious integration model. Serial ctDNA may improve risk awareness and timing of reassessment, but routine clinical decisions should still be anchored in clinical status, imaging, CA19–9 context, and multidisciplinary judgment until prospective trials define how to act on molecular progression or MRD positivity (19, 20, 22).

### 4.3 Methodologic heterogeneity and standardization challenges

The heterogeneity observed across studies is not only a limitation of evidence synthesis but also a barrier to clinical interpretation, because the same qualitative ctDNA pattern may have different meanings depending on assay sensitivity, sampling window, disease burden, and treatment context. Differences in assay design, reporting metrics, and sampling schedules made direct comparison across PDAC ctDNA studies difficult and limited the suitability of pooled quantitative synthesis. Included studies used both tumor-informed approaches (leveraging tumor tissue to define patient-specific targets and reporting outputs such as MTM/mL within prespecified MRD and surveillance windows) and tumor-agnostic approaches (broad sequencing without a tumor-informed search, often relying on error-suppression strategies and/or leukocyte controls) (3, 4). In parallel, platforms ranged from PCR-based KRAS assays to targeted NGS and broader sequencing strategies, each with different limits of detection, gene content, and susceptibility to false positives from clonal hematopoiesis, which can materially influence apparent baseline positivity rates and longitudinal clearance definitions (2, 5, 17). Clonal hematopoiesis of indeterminate potential (CHIP) is particularly relevant for tumor-agnostic NGS assays because age-related hematopoietic clones can release variants into plasma that may be mistaken for tumor-derived alterations. This is especially important when variants occur in genes commonly affected by CHIP or when plasma-only sequencing is performed without matched leukocyte DNA. In serial monitoring studies, CHIP-related variants may inflate baseline ctDNA positivity, obscure true molecular clearance, or be misclassified as molecular progression if variant origin is not carefully adjudicated. By contrast, tumor-informed assays that track patient-specific tumor variants may reduce, but not completely eliminate, this risk, depending on tissue quality, variant selection, and bioinformatic filtering. Future PDAC ctDNA studies should therefore report whether matched leukocyte DNA, tumor tissue confirmation, or CHIP-filtering algorithms were used and should distinguish tumor-derived variants from probable hematopoietic variants when defining ctDNA positivity, clearance, or progression (4, 13, 20).

Heterogeneity in quantification and reporting units further complicates translation. Studies variably reported ctDNA as VAF/MAF, copies, ddCq-based measures, or MTM/mL, and applied different thresholds for positivity and response classification. Consequently, a decline or clearance event in one platform may not correspond to an equivalent biologic or clinical change in another, limiting the ability to define universal thresholds for molecular response or molecular progression (3, 6, 11).

Sampling schedules also differed substantially, both in frequency and in alignment with clinical landmarks. Some cohorts used structured perioperative windows (e.g., defined postoperative MRD timeframes and surveillance intervals), whereas others obtained samples opportunistically during routine visits, and imaging intervals ranged from tightly scheduled to clinician driven. This mismatch in cadence can bias apparent lead-time estimates and may partly explain differences in reported concordance with imaging and CA19–9 across studies (1, 2, 16).

Finally, variability in cohort composition and analytic adjustment interacts with methodologic heterogeneity. Mixing disease stages, treatment lines, or metastatic patterns without consistent stratification, and using different covariate sets for multivariable models, can amplify between-study differences even when assays are similar. Future studies should use predefined perioperative sampling windows, prespecified monitoring intervals in advanced disease, and consistent definitions of ctDNA positivity, clearance, and clinically actionable change (7, 15).

Standardization should begin at the pre-analytic and analytic levels. Future studies should clearly report specimen type, blood collection tubes, processing time, plasma volume, cfDNA input, sequencing depth or PCR sensitivity, variant calling criteria, lower limit of detection, lower limit of quantification when available, and whether matched leukocyte DNA was used to reduce false-positive findings from clonal hematopoiesis. Assay sensitivity should also be interpreted in relation to clinical context: a threshold suitable for high-burden metastatic disease may not be adequate for postoperative MRD detection, where ctDNA levels are expected to be low. Therefore, analytical thresholds should be prespecified and reported separately for baseline detection, MRD positivity, molecular response, and molecular progression (19, 20, 22).

Reporting units also require harmonization. Included studies variably reported ctDNA as VAF, MAF, copies/mL, ddCq-based measures, MTM/mL, or binary positive/negative status, limiting direct comparison across platforms. Future reports should present both qualitative status and quantitative ctDNA burden when possible, specify the denominator used for variant quantification, and define whether molecular clearance refers to complete undetectability, decline below a platform-specific threshold, or a prespecified percentage reduction from baseline. For longitudinal monitoring, studies should distinguish positive-to-negative conversion, persistent negativity, persistent positivity, molecular response, and molecular progression, because these patterns may carry different prognostic and clinical implications (3, 5, 6, 11, 15, 20).

MRD definitions and sampling intervals should also be standardized by clinical setting. In resected PDAC, postoperative MRD assessment should use prespecified windows, such as early postoperative, post-adjuvant, and surveillance timepoints, and should account for perioperative cfDNA fluctuations that may affect test interpretation. In locally advanced or metastatic disease, ctDNA sampling should be aligned with treatment cycles and imaging schedules to reduce bias in estimating lead time over radiographic progression. Clinically actionable response criteria should be defined prospectively, including the magnitude of ctDNA decline considered meaningful, the number of consecutive positive samples required to define molecular progression, and the recommended action for discordant results such as rising ctDNA with stable imaging or falling ctDNA with rising CA19–9 (2, 3, 5, 11, 15, 20).

Finally, standardization should extend to outcome reporting. Studies should consistently report whether ctDNA endpoints are exploratory or prespecified, whether analyses are adjusted for tumor burden, stage, treatment regimen, CA19-9, and imaging frequency, and whether ctDNA-guided actions were performed. Adoption of harmonized prognostic biomarker reporting frameworks, such as REMARK, together with ctDNA-specific reporting of analytical validity, clinical validity, and clinical utility, would improve comparability across PDAC studies and facilitate translation into interventional trials (19, 20, 22).

### 4.4 Quality of evidence and bias considerations

Evidence for serial ctDNA in PDAC is limited mainly by observational study designs and differences in analytic methods across studies. Using the QUIPS framework, most included studies were judged to have moderate risk of bias, with fewer studies at high risk, reflecting recurring limitations in cohort selection, incomplete longitudinal sampling, and inconsistent control for confounding. These issues are particularly important in PDAC because ctDNA detectability and kinetics are strongly influenced by tumor burden, metastatic pattern, treatment regimen/line of therapy, and timing of sampling relative to interventions, all of which can bias associations with survival or recurrence if not adequately adjusted.

Selection bias was common because many cohorts were drawn from single institutions or tertiary referral settings, and several studies required availability of tumor tissue, sufficient plasma volume, or successful genomic profiling, which may enrich for patients with specific disease characteristics. Attrition and missingness were also non-trivial: longitudinal designs frequently had variable numbers of samples per patient and incomplete follow-up timepoints, potentially biasing analyses if missingness was related to clinical deterioration or early death.

Measurement bias remains a central concern given heterogeneity in ctDNA platforms, positivity thresholds, and reporting units, as well as potential false positives from clonal hematopoiesis in panel-based sequencing when leukocyte controls are not incorporated. While most studies used standard outcome definitions (OS, PFS, DFS/RFS) and radiographic assessment frameworks, imaging frequency and adjudication practices varied, which can influence both progression dating and lead-time estimates. In addition, selective reporting is possible because studies may preferentially highlight timepoints or ctDNA metrics that show stronger associations, particularly when multiple assays, targets, or thresholds were explored.

Taken together, these limitations support a cautious interpretation: current evidence most strongly supports the prognostic value of ctDNA, especially postoperative MRD, yet the magnitude and actionability of effects likely vary by assay, timing, and clinical context. Future studies should prespecify sampling schedules and ctDNA endpoints, adjust for tumor burden and treatment factors, and test whether ctDNA-guided decisions improve outcomes in prospective designs.

### 4.5 Implementation considerations and precision oncology framework

Clinical implementation of serial ctDNA in PDAC should be considered within a biomarker-development and precision oncology framework. A key distinction is whether ctDNA is being used as a prognostic, monitoring, or predictive biomarker. In the studies included in this review, serial ctDNA most consistently functioned as a prognostic and disease-monitoring biomarker: postoperative ctDNA positivity identified patients at higher risk of relapse, while on-treatment ctDNA persistence or rise was associated with progression and inferior survival (2, 3, 5, 6, 11, 15). By contrast, a predictive biomarker should identify patients more or less likely to benefit from a specific intervention. Most available PDAC ctDNA studies did not prospectively assign therapy based on ctDNA status or test whether ctDNA dynamics modify treatment benefit. Therefore, ctDNA in PDAC should currently be viewed primarily as a risk-stratification and monitoring tool, whereas its predictive role for treatment selection remains investigational (19, 20, 22).

From a precision oncology perspective, serial ctDNA may eventually support several clinically meaningful workflows. In resected or localized PDAC, postoperative MRD positivity could identify patients at high risk for recurrence who may benefit from intensified surveillance, earlier imaging, enrollment in ctDNA-directed clinical trials, or future studies of adjuvant treatment escalation (2, 3, 14, 18). Conversely, persistent postoperative ctDNA negativity may eventually help identify lower-risk patients for de-escalation strategies, although this remains investigational. In locally advanced or metastatic PDAC, early ctDNA decline or clearance during systemic therapy may support continued treatment when concordant with clinical status, imaging, and CA19-9, whereas persistent or rising ctDNA may prompt earlier radiographic reassessment, closer surveillance, evaluation for resistance mechanisms, or clinical trial consideration (5, 6, 11, 15, 17). Importantly, treatment switching or escalation based solely on ctDNA dynamics should not be adopted routinely until prospective interventional studies demonstrate that ctDNA-guided decisions improve patient outcomes rather than only detecting poor-risk disease earlier (20).

Clinical use of serial ctDNA in PDAC will depend on practical issues that differ between resected and advanced disease. In localized disease, tumor-informed MRD strategies depend on timely access to tumor tissue and a workflow that can generate a patient-specific assay within a clinically meaningful postoperative window; this is reflected in studies that define prespecified MRD/surveillance windows and rely on tumor-informed personalization (e.g., Signatera-based frameworks) (3). Tumor-agnostic approaches may improve feasibility by reducing dependence on tissue, but they typically require robust error-suppression methods to maintain specificity, as illustrated by tumor-agnostic NGS strategies used without a tumor-informed search (4). In addition, some NGS frameworks explicitly address false positives by excluding variants attributable to clonal hematopoiesis using matched blood components, underscoring the practical importance of such controls for implementation (13). In clinical workflows, this is particularly important when ctDNA results are discordant with imaging and CA19-9, because a low-level plasma variant of hematopoietic origin could otherwise prompt unnecessary surveillance escalation, repeat imaging, or inappropriate treatment concern.

Recent advances in liquid biopsy further emphasize that assay choice should be matched to the intended clinical use. Tumor-informed assays may be particularly attractive for postoperative MRD detection because they track patient-specific tumor variants, whereas tumor-agnostic approaches may be more feasible when tumor tissue is unavailable but may require broader sequencing, error suppression, and clonal hematopoiesis filtering to preserve specificity (3, 4, 13, 17, 25). Contemporary reviews of ctDNA implementation in pancreatobiliary tumors similarly highlight the evolution from ddPCR-based mutation tracking to NGS-based tumor-informed and tumor-agnostic assays, while emphasizing persistent barriers such as low ctDNA yield, stromal desmoplasia, clonal hematopoiesis, assay standardization, cost, and uncertain clinical utility (25).

Pre-analytic and analytic variables also influence implementation. Differences in sample type, processing, and assay-specific limits of detection can affect detectability and comparability across timepoints, particularly when perioperative sampling is used. Studies implementing complex perioperative sampling designs, including paired portal and peripheral blood, plus structured postoperative schedules, highlight how sampling strategy itself can shape feasibility and interpretability (1). For longitudinal monitoring, feasibility is closely tied to sampling cadence; existing cohorts demonstrate a wide range of monitoring intervals (e.g., every 4 weeks during therapy, every 4–8 weeks, or every 2 months depending on clinical workflow and platform), which has direct implications for real-world scalability (5, 11, 17). These implementation challenges underscore the need for a minimum ctDNA reporting dataset that includes assay platform, tumor-informed status, gene targets, sensitivity, limit of detection, reporting unit, sampling window, imaging interval, CA19–9 context, and prespecified criteria for molecular response or progression (19, 20, 22, 25).

Clinical integration also depends on defining actionable change thresholds and workflows for discordant results. Many included studies interpreted ctDNA alongside imaging and CA19-9, supporting the practicality of integrating ctDNA into existing monitoring rather than replacing standard assessments (5, 6, 11, 15). In practice, concordant changes in ctDNA, imaging, and CA19–9 may increase confidence in response or progression, while discordant findings, such as rising ctDNA with stable imaging, may prompt earlier repeat imaging or closer surveillance (2, 5). However, escalation based solely on ctDNA remains investigational and requires prospective evaluation to avoid overtreatment.

Translation into routine practice requires evidence across three levels: analytical validity, clinical validity, and clinical utility. Analytical validity requires reliable detection of low-frequency variants across pre-analytic conditions, platforms, and reporting units. Clinical validity requires consistent association between ctDNA findings and clinically meaningful outcomes such as recurrence, PFS, OS, or radiographic progression. Clinical utility requires evidence that acting on ctDNA results improves outcomes, reduces unnecessary treatment, improves quality of life, or provides cost-effective care compared with standard management. The current PDAC literature provides growing evidence for clinical validity, particularly for postoperative MRD and on-treatment monitoring, but evidence for clinical utility remains limited because few studies have prospectively tested ctDNA-triggered management strategies (19, 20, 22).

Finally, health-system factors, including assay availability, turnaround time, reimbursement, cost-effectiveness, accessibility, and laboratory standardization, will strongly influence adoption. Tumor-informed assays may require tumor tissue acquisition, sequencing, assay design, and additional processing time before results can inform postoperative MRD decisions, whereas tumor-agnostic assays may offer faster deployment but can be affected by lower specificity, clonal hematopoiesis, and platform-dependent reporting thresholds. Serial monitoring also creates cumulative cost considerations because repeated blood draws, sequencing, molecular interpretation, and follow-up imaging may be required over time. Therefore, implementation will depend on whether ctDNA testing provides sufficient clinical value to justify repeated testing, particularly if ctDNA results trigger earlier imaging, intensified surveillance, or treatment changes. Reimbursement policies, institutional access to validated assays, turnaround time compatible with clinical decision-making, and equitable availability across community and academic settings will be important determinants of real-world use. Although some studies used commercially available assays or multicenter testing, prospective trials and health-economic analyses are still needed to define sampling windows, thresholds, ctDNA-triggered actions, and whether these strategies improve outcomes in a cost-effective manner (3, 17, 20).

### 4.6 Future research agenda

Future research should determine whether serial ctDNA can move beyond prognostic assessment and guide clinical decisions in PDAC. First, studies should adopt harmonized reporting and design standards for prognostic biomarkers, including prespecified hypotheses, transparent assay methods, complete reporting of analytical thresholds, standardized ctDNA reporting units, and complete reporting of all planned timepoints and outcomes, to improve reproducibility and interpretability across cohorts (REMARK) (19, 22). For serial ctDNA specifically, future protocols should define MRD positivity, molecular response, molecular clearance, and molecular progression before analysis, and should specify how these endpoints will be interpreted alongside CA19–9 and imaging (20). Second, prospective, stage-stratified protocols are needed with prespecified sampling windows (baseline, early postoperative MRD windows, post-adjuvant, and surveillance) and structured on-treatment schedules aligned to imaging intervals, reflecting the types of perioperative and longitudinal frameworks already used in the PDAC ctDNA literature (e.g., landmark MRD/surveillance windows and serial monitoring during chemotherapy). Third, head-to-head comparisons should directly test tumor-informed versus tumor-agnostic strategies and ddPCR/dPCR versus targeted NGS approaches, while standardizing definitions for positivity, clearance, molecular progression, and clinically actionable change. Recent liquid biopsy literature emphasizes that tumor-informed assays, tumor-agnostic panels, methylation-based approaches, fragmentomics, and multi-omic strategies may have different strengths depending on whether the clinical goal is postoperative MRD detection, early recurrence identification, treatment-response monitoring, or resistance profiling (24–26). Therefore, future PDAC studies should prespecify the intended clinical use of ctDNA before selecting the assay platform and sampling schedule. Fourth, interventional trials are needed to determine whether ctDNA-guided actions, such as ctDNA-triggered early imaging, escalation or de-escalation of adjuvant therapy, or therapy switching upon molecular progression, improve outcomes beyond earlier detection alone. This distinction is central to ctDNA implementation because clinical validity does not necessarily establish clinical utility; a biomarker may predict recurrence or progression without yet proving that acting on the result improves survival, quality of life, or treatment value (20). Future studies should also control for key confounders, use standardized risk-of-bias assessment such as QUIPS, and prespecify subgroup analyses, including borderline resectable/NAT cohorts, patients with normal CA19-9, low tumor burden, and different metastatic patterns (21). Finally, future ctDNA-guided PDAC trials should be designed within biomarker-guided precision oncology frameworks that distinguish biomarker discovery from clinical qualification, require independent validation, and test whether biomarker-directed actions improve patient outcomes rather than simply identifying high-risk disease earlier (19, 20, 22, 26).

### 4.7 Strengths and limitations

This review has several strengths. It focuses on serial ctDNA dynamics in PDAC rather than single-timepoint detection, addressing molecular response, molecular progression, and postoperative MRD surveillance across disease stages. Study selection and data extraction captured outcomes, assay platforms, and sampling schedules, allowing findings to be interpreted across tumor-informed and tumor-agnostic approaches and across PCR- and NGS-based assays (3, 4). Study quality was appraised using a prognosis-focused risk-of-bias framework (QUIPS), supporting a transparent assessment of internal validity and interpretation of findings across cohorts (21).

Several limitations should be considered. First, the evidence base is dominated by observational cohort studies, many of which are single-center and include relatively small sample sizes, increasing the risk of selection bias and imprecision. Second, substantial heterogeneity in ctDNA assays (tumor-informed vs tumor-agnostic; PCR vs NGS), reporting metrics (e.g., VAF/MAF, copies, MTM/mL), and sampling schedules limited direct cross-study comparability and precluded meta-analysis (3, 6, 11). Although random-effects meta-analysis can account for statistical heterogeneity, it cannot resolve non-comparable biomarker definitions, inconsistent sampling windows, and clinically distinct outcome constructs; therefore, narrative synthesis was selected to avoid generating potentially misleading pooled estimates. In addition, combining studies across resected, localized, borderline resectable, locally advanced, and metastatic PDAC may introduce clinical heterogeneity; therefore, conclusions were framed by clinical use-case rather than as single generalized estimate across all disease stages. Third, confounding remains a key concern, as ctDNA kinetics are influenced by tumor burden, metastatic patterns, treatment regimens, and timing of sampling, and adjustment strategies were inconsistent across studies (5, 7). Fourth, attrition and missing longitudinal samples were common, which may bias associations if missingness is related to clinical deterioration. Another limitation is that PubMed/MEDLINE was the only comprehensive bibliographic database searched, supplemented by targeted journal/publisher platform searches and manual reference-list screening. Embase, Scopus, Web of Science, the Cochrane Library, and ClinicalTrials.gov were not searched. This may increase the risk of missing relevant studies, particularly given inconsistent terminology across ctDNA platforms and the rapid evolution of liquid biopsy research. However, this review was restricted to peer-reviewed, full-text original studies with serial ctDNA assessment and clinically meaningful PDAC-specific outcomes; therefore, unpublished trials, protocols, and ongoing studies identified through trial registries would not have met the prespecified eligibility criteria. Finally, publication and selective reporting bias are possible because studies may preferentially report timepoints or ctDNA metrics with stronger associations, and few studies tested ctDNA-guided management strategies prospectively. These limitations also lowered the certainty of evidence, particularly for on-treatment monitoring, lead-time estimation, and ctDNA-guided treatment decision-making.

### 4.8 ctDNA within a multimodal biomarker landscape

Although CA19–9 and imaging are the most commonly available comparators, ctDNA should not be viewed as the only emerging biomarker in PDAC. Instead, serial ctDNA kinetics are likely to be most informative when incorporated into composite biomarker models that combine tumor-derived genomic information, circulating protein or immune biomarkers, radiographic/radiomic features, and tissue-based spatial or immune profiling. In the studies included in this review, ctDNA was frequently interpreted alongside imaging and CA19–9 (5, 6, 11, 15), while tumor-informed and NGS-based approaches linked plasma findings to tumor molecular profiles (3, 4, 13, 17). Future models could combine ctDNA positivity, quantitative ctDNA burden or clearance, CA19–9 kinetics, radiographic tumor burden, molecular alterations, and clinical covariates such as disease stage, treatment line, and metastatic pattern to improve risk stratification beyond any single marker (7, 15, 19, 20).

Recent PDAC biomarker literature supports this multimodal direction. Yu et al. highlighted emerging biomarkers, including miRNAs and ctDNA, as well as the role of the tumor microenvironment, immune-suppressive cells, cancer-associated fibroblasts, extracellular matrix remodeling, and AI-driven multi-omics analysis in personalized PDAC care (24). Immune and spatial biomarkers may be particularly relevant because PDAC biology is shaped by an immunosuppressive and stromal-rich microenvironment. Measures of immune-cell composition, including the balance between effector T cells and regulatory T cells, may provide complementary information about immune competence, tumor immune evasion, and potential responsiveness to immunomodulatory strategies. The effector-to-regulatory T-cell ratio has recently been highlighted as a biomarker framework with prognostic and therapeutic relevance in pancreatic cancer, with lower Teff/Treg ratios associated with poor clinical outcomes, tumor progression, and immunotherapy resistance (23).

Similarly, recent pancreatobiliary ctDNA reviews emphasize that ctDNA-guided strategies are likely to be most useful when integrated with clinical risk factors, CA19–9 kinetics, imaging, multi-omics data, and AI-assisted signal interpretation rather than interpreted as isolated binary results (25). Integration of ctDNA with immune-cell phenotyping, spatial tissue profiling, transcriptomic or proteomic signatures, and imaging-derived features may therefore help define biologically distinct patient subgroups and support rational clinical trial design (23–26).

However, multimodal biomarker models must undergo the same validation pathway as ctDNA alone: analytical validity, clinical validity, and clinical utility. Composite scores should be developed using prespecified variables, validated in independent PDAC cohorts, and tested prospectively to determine whether biomarker-guided interventions improve outcomes compared with standard CA19-9- and imaging-based management (19, 20, 22). Until such evidence is available, multimodal biomarker integration should be considered a translational research priority rather than a routine clinical decision tool.

## 5 Conclusion

Across clinically distinct PDAC settings, serial ctDNA assessment was associated with recurrence, progression, treatment response, and survival, but its interpretation differs by disease stage and treatment context. The strongest evidence supports postoperative ctDNA as an MRD marker in resected or localized PDAC, where ctDNA positivity identifies patients at higher relapse risk and ctDNA negativity or clearance is associated with lower recurrence risk. In locally advanced and metastatic PDAC, on-treatment ctDNA changes may help identify response or progression and may add information to imaging and CA19-9. However, differences in assays, reporting units, sampling schedules, and confounder adjustment limit the definition of actionable thresholds. Prospective studies, including interventional trials with standardized assays, prespecified MRD windows, harmonized reporting units, defined molecular response/progression criteria, and ctDNA-triggered clinical actions, are needed before ctDNA-guided management can be adopted routinely.

## Data Availability

The original contributions presented in the study are included in the article/**Supplementary Material**. Further inquiries can be directed to the corresponding author.

## References

[1] Maulat C Canivet C Cabarrou B Pradines A Selves J Casanova A . Prognostic impact of circulating tumor DNA detection in portal and peripheral blood in resected pancreatic ductal adenocarcinoma patients. Sci Rep. (2024) 14:15–6. doi: 10.1038/s41598-024-76903-y 39516243 PMC11549393

[2] Groot VP Mosier S Javed AA Teinor JA Gemenetzis G Ding D . Circulating tumor DNA as a clinical test in resected pancreatic cancer. Clin Cancer Research: Off J Am Assoc For Cancer Res. (2019) 25:4973–84. doi: 10.1158/1078-0432.CCR-19-0197 31142500 PMC7403524

[3] Botta GP Abdelrahim M Drengler RL Aushev VN Esmail A Laliotis G . Association of personalized and tumor-informed ctDNA with patient survival outcomes in pancreatic adenocarcinoma. Oncologist. (2024) 29:859–69. doi: 10.1093/oncolo/oyae155 39022993 PMC11449101

[4] Affolter KE Hellwig S Nix DA Bronner MP Thomas A Fuertes CL . Detection of circulating tumor DNA without a tumor-informed search using next-generation sequencing is a prognostic biomarker in pancreatic ductal adenocarcinoma. Neoplasia (New York NY). (2021) 23:859–69. doi: 10.1016/j.neo.2021.06.005 34298235 PMC8322473

[5] Edland KH Tjensvoll K Oltedal S Dalen I Lapin M Garresori H . Monitoring of circulating tumour DNA in advanced pancreatic ductal adenocarcinoma predicts clinical outcome and reveals disease progression earlier than radiological imaging. Mol Oncol. (2023) 17:1857–70. doi: 10.1002/1878-0261.13472 37341038 PMC10483602

[6] Chen I Raymond VM Geis JA Collisson EA Jensen BV Hermann KL . Ultrasensitive plasma ctDNA KRAS assay for detection, prognosis, and assessment of therapeutic response in patients with unresectable pancreatic ductal adenocarcinoma. Oncotarget. (2017) 8:97769–86. doi: 10.18632/oncotarget.22080 29228650 PMC5716690

[7] Strijker M Soer EC de Pastena M Creemers A Balduzzi A Beagan JJ . Circulating tumor DNA quantity is related to tumor volume and both predict survival in metastatic pancreatic ductal adenocarcinoma. Int J Cancer. (2020) 146:1445–56. doi: 10.1002/ijc.32586 31340061 PMC7004068

[8] Page MJ McKenzie JE Bossuyt PM Boutron I Hoffmann TC Mulrow CD . The PRISMA 2020 statement: an updated guideline for reporting systematic reviews. BMJ. (2021) 372:n71. doi: 10.31222/osf.io/v7gm2_v1 33782057 PMC8005924

[9] Huerta M Martín-Arana J Gimeno-Valiente F Carbonell-Asins JA García-Micó B Martínez-Castedo B . CtDNA whole exome sequencing in pancreatic ductal adenocarcinoma unveils organ-dependent metastatic mechanisms and identifies actionable alterations in fast progressing patients. Trans Res. (2024) 271:105–15. doi: 10.1016/j.trsl.2024.05.003 38782356

[10] Aaquist T Henriksen TV Mariager Jakobsen LL Fristrup CW Pfeiffer P de Stricker K . Circulating tumour DNA as a prognostic tool for surgically treated pancreatic ductal adenocarcinoma. Hum Pathol. (2025) 168:106024. doi: 10.1016/j.humpath.2025.106024 41421722

[11] Sugimori M Sugimori K Tsuchiya H Suzuki Y Tsuyuki S Kaneta Y . Quantitative monitoring of circulating tumor DNA in patients with advanced pancreatic cancer undergoing chemotherapy. Cancer Sci. (2020) 111:266–78. doi: 10.1111/cas.14245 31746520 PMC6942439

[12] Labiano I Huerta AE Alsina M Arasanz H Castro N Mendaza S . Building on the clinical applicability of ctDNA analysis in non-metastatic pancreatic ductal adenocarcinoma. Sci Rep. (2024) 14:16203. doi: 10.1038/s41598-024-67235-y 39003322 PMC11246447

[13] Murakami T Imamura M Kimura Y Watanabe K Shinohara Y Nakamura T . Role of preoperative circulating tumor DNA in predicting occult metastases in resectable and borderline resectable pancreatic ductal adenocarcinoma. World J Gastroenterol. (2025) 31:109383. doi: 10.3748/wjg.v31.i32.109383 40900777 PMC12400235

[14] Lee B Lipton L Cohen J Tie J Javed AA Li L . Circulating tumor DNA as a potential marker of adjuvant chemotherapy benefit following surgery for localized pancreatic cancer. Ann Oncol Off J Eur Soc For Med Oncol. (2019) 30:1472–8. doi: 10.1093/annonc/mdz200 31250894 PMC6771221

[15] Kim H Lee J Park MR Choi Z Han SJ Kim D . Prognostic value of residual circulating tumor DNA in metastatic pancreatic ductal adenocarcinoma. Ann Lab Med. (2025) 45:199–208. doi: 10.3343/alm.2024.0345 39801270 PMC11788705

[16] Perets R Greenberg O Shentzer T Semenisty V Epelbaum R Bick T . Mutant KRAS circulating tumor DNA is an accurate tool for pancreatic cancer monitoring. Oncologist. (2018) 23:566–72. doi: 10.1634/theoncologist.2017-0467 29371474 PMC5947453

[17] Mahadevia H Majeed U Patel J Ahmed AK Elhariri A Albelal D . Circulating tumor DNA and tissue testing for pancreatobiliary tumors. JAMA Netw Open. (2025) 8:e2531373. doi: 10.1001/jamanetworkopen.2025.31373 40938601 PMC12432637

[18] Jiang J Ye S Xu Y Chang L Hu X Ru G . Circulating tumor DNA as a potential marker to detect minimal residual disease and predict recurrence in pancreatic cancer. Front Oncol. (2020) 10:1220. doi: 10.3389/fonc.2020.01220 32850360 PMC7406781

[19] Altman DG McShane LM Sauerbrei W . Reporting recommendations for tumor marker prognostic studies (REMARK): explanation and elaboration. PloS Med. (2012) 9:e1001216. doi: 10.1371/journal.pmed.1001216 22675273 PMC3362085

[20] Pascual J Attard G Bidard FC Curigliano G de Mattos-Arruda L Diehn M . ESMO recommendations on the use of circulating tumour DNA assays for patients with cancer: a report from the ESMO Precision Medicine Working Group. Ann Oncol Off J Eur Soc For Med Oncol. (2022) 33:750–68. doi: 10.1016/j.annonc.2022.05.520 35809752

[21] Hayden JA van der Windt DA Cartwright JL Côté P Bombardier C . Assessing bias in studies of prognostic factors. Ann Internal Med. (2013) 158:280–6. doi: 10.7326/0003-4819-158-4-201302190-00009 23420236

[22] McShane LM Altman DG Sauerbrei W Taube SE Gion M Clark GM . Reporting recommendations for tumor marker prognostic studies (REMARK). J Natl Cancer Institute. (2005) 97:1180–4. doi: 10.1093/jnci/dji237 16106022

[23] Guo X Zhang H Yao L Ma W . Harnessing the effector-to-regulatory T cell ratio to advance prognosis and precision therapy in pancreatic cancer. Crit Rev Oncology/Hematology. (2026) 218:105076. doi: 10.1016/j.critrevonc.2025.105076 41380868

[24] Yu B Shao S Ma W . Frontiers in pancreatic cancer on biomarkers, microenvironment, and immunotherapy. Cancer Lett. (2025) 610:217350. doi: 10.1016/j.canlet.2024.217350 39581219

[25] Liu R Yang Y Hu Z Fu Y . Circulating tumor DNA-guided early detection and minimal residual disease monitoring in pancreatic and biliary cancers: evidence, barriers, and opportunities. World J Surg Oncol. (2026) 24:114. doi: 10.1186/s12957-026-04238-1 41652428 PMC12973717

[26] Bartolomucci A Nobrega M Ferrier T Dickinson K Kaorey N Nadeau A . Circulating tumor DNA to monitor treatment response in solid tumors and advance precision oncology. NPJ Precis Oncol. (2025) 9:84. doi: 10.1038/s41698-025-00876-y 40122951 PMC11930993

