# Correction: Serial ctDNA dynamics predict clinical outcomes in metastatic and locally advanced PDAC: a systematic review

**DOI:** 10.3389/fonc.2026.1955091

**Published:** 2026-08-12

**Authors:** Supriya Peshin, Ehab Takrori, Ibrahim Halil Sahin, Harrison Kim, Mark Rubinstein, Claire Verschragen, Zahra Hamedi, Shafia Rahman

**Affiliations:** 1 Department of Internal Medicine, Norton Community Hospital, Norton, VA, United States; 2 College of Medicine, Alfaisal University, Riyadh, Saudi Arabia; 3 Department of Gastrointestinal Medical Oncology, University of Michigan, Ann Arbor, MI, United States; 4 Department of Radiology, The Ohio State University, Columbus, OH, United States; 5 Pelotonia Institute for Immuno-Oncology, The Ohio State University Comprehensive Cancer Center-James Cancer Center and Solove Research Institute, Columbus, OH, United States; 6 Division of Medical Oncology, The Ohio State University Comprehensive Cancer Center, Columbus, OH, United States; 7 Division of Hematology and Oncology, Department of Medicine, University of California Irvine, Orange, CA, United States

**Keywords:** CA19-9, circulating tumor DNA, early molecular response, liquid biopsy, longitudinal monitoring, minimal residual disease, overall survival, pancreatic ductal adenocarcinoma

Affiliation 7 Department of Gastrointestinal Medical Oncology, University of California, Irvine, Irvine, CA, United States was erroneously assigned to Dr. Zahra Hamedi. This affiliation has now been removed.

The original version of this article has been updated.

