# Supplementary material for: Serial ctDNA dynamics predict clinical outcomes in metastatic and locally advanced PDAC: a systematic review
Source: Front Oncol. 2026 Jul 30;16:1901721. doi: 10.3389/fonc.2026.1901721 (PMC13508053; doi:10.3389/fonc.2026.1901721)
Supplement: Supplementary file 1 [file Table1.docx]

Supplementary Table S1. Detailed Methodological Characteristics of Included Studies Evaluating Serial ctDNA Dynamics in PDAC

| **Study** | **Design** | **Setting / Stage** | **ctDNA platform / approach** | **Sampling timepoints** | **Primary outcomes / clinical focus** |
| --- | --- | --- | --- | --- | --- |
| Huerta 2024 | Prospective cohort | Metastatic PDAC | Plasma WES + ddPCR KRAS | Baseline then q2-3 months aligned with CT; validation: baseline + first response evaluation | PFS/OS; ctDNA detectability + VAF change; imaging/CA19-9 comparisons |
| Mahadevia 2025 | Retrospective cohort | Advanced PDAC | Guardant360 ctDNA NGS | Serial ctDNA q2 months; concordance window ≤90 days for tissue vs ctDNA | Tissue-ctDNA concordance; response monitoring vs RECIST and CA19-9 |
| Aaquist 2025 | Observational multicenter cohort | Resected PDAC | Tumor-informed (tumor NGS single mutation) + ddPCR | Pre-op; 1 month post-op; 7-9 months post-op | OS/RFS; ctDNA ± CA19-9 |
| Affolter 2021 | Observational perioperative cohort | Surgical PDAC + Healthy Controls | Tumor-uninformed NGS | Pre-op; post-op within 100 days | Survival association of perioperative ctDNA detection |
| Botta 2024 | Retrospective cohort | Survival analysis in resected stage I-III | Tumor-informed personalized mPCR-NGS (Signatera; MTM/mL) | Diagnosis; MRD window 2-12 weeks post-op; surveillance >12 weeks | DFS (radiologic recurrence); ctDNA MRD/surveillance prognostic modeling including CA19-9 |
| Chen 2017 | Prospective cohort | Unresectable PDAC (stage III-IV) | KRAS-mutation enrichment PCR + NGS (MiSeq) | Baseline; pre-cycle 2; then with CT assessments | TTP/OS; longitudinal ctDNA vs RECIST and CA19-9 |
| Edland 2023 | Prospective cohort | Advanced/inoperable PDAC | KRAS PNA clamp qPCR | Baseline; q4 weeks during chemotherapy; CT q8 weeks | PFS/OS; earlier progression detection vs imaging; CA19-9 comparator |
| Groot 2019 | Prospective cohort | Resected/localized PDAC | ddPCR KRAS (RainDrop); tumor KRAS by tumor NGS | Pre-incision; discharge/1 month; then q2-3 months | RFS/OS; recurrence prediction + lead-time vs imaging |
| Jiang 2020 | Cohort | Surgical PDAC | Hybrid capture NGS; VAF | Pre-op; POD7; then 1-3 months post-op | DFS/recurrence prediction (MRD paradigm); CA19-9/CT as comparators |
| Kim 2025 | Cohort | Metastatic PDAC | Targeted NGS | Baseline; response-evaluation timepoint during therapy | PFS/OS by baseline and residual ctDNA; CA19-9 comparator |
| Labiano 2024 | Prospective cohort | Non-metastatic PDAC | Oncomine cfDNA NGS + tumor NGS | Diagnosis; first assessment after NAT; clinical progression | OS/EFS; ctDNA dynamics across NAT course; radiology/CA19-9 context |
| Lee 2019 | Prospective multicenter biomarker trial | Localized/resectable PDAC | SafeSeqS ctDNA; tumor KRAS hotspot assay | Pre-op; 4-8 weeks post-op | RFS/OS; ctDNA as marker of adjuvant benefit; RECIST/CA19-9 |
| Maulat 2024 | Prospective cohort | Resected PDAC | Tumor NGS + ddPCR KRAS | Intraoperative portal + peripheral; POD1/10; POM1/3/6/12 | ctDNA compartment kinetics; RFS/OS; CA19-9 + recurrence assessment |
| Murakami 2025 | Prospective cohort | Resectable/borderline PDAC | Oncomine cfDNA targeted NGS | Day of surgery; subset also at NAT start | Occult metastasis prediction; RFS/OS; CA19-9 + imaging surveillance |
| Perets 2018 | Cohort | Metastatic PDAC | Targeted KRAS sequencing (Ion Torrent PGM) | Serial (each follow-up visit); 1-8 samples; ≥4 weeks apart | Monitoring accuracy; ctDNA dynamics vs CA19-9 and CT imaging; survival association |
| Strijker 2020 | Retrospective analysis of prospectively collected samples | Treatment-naïve metastatic PDAC | Targeted NGS (amplicon panel) + ddPCR validation | Baseline (for all); follow-up at response evaluation (for subset) | OS; ctDNA quantity vs tumor volume; CA19-9 context |
| Sugimori 2020 | Prospective multicenter cohort | Advanced PDAC | dPCR KRAS (±NGS for non-G12/13); MAF | Baseline; monitoring subset q4-8 weeks during chemotherapy | Treatment monitoring; ctDNA dynamics vs RECIST and tumor markers (CA19-9/CEA) |

*Abbreviations:* PDAC, pancreatic ductal adenocarcinoma; WES, whole-exome sequencing; ddPCR, digital droplet polymerase chain reaction; KRAS, Kirsten rat sarcoma viral oncogene homolog; PFS, progression-free survival; OS, overall survival; ctDNA, circulating tumor DNA; VAF, variant allele fraction; NGS, next-generation sequencing; RFS, recurrence-free survival; mPCR, multiplex polymerase chain reaction; MTM/mL, mean tumor molecules per milliliter; MRD, minimal residual disease; DFS, disease-free survival; PCR, polymerase chain reaction; MiSeq, Illumina MiSeq sequencing platform; TTP, time to progression; PNA clamp, peptide nucleic acid clamp; qPCR, quantitative polymerase chain reaction; POD, postoperative day; cfDNA, cell-free DNA; NAT, neoadjuvant therapy; EFS, event-free survival; SafeSeqS, Safe-Sequencing System; POM, postoperative month; PGM, Personal Genome Machine (Ion Torrent); dPCR, digital polymerase chain reaction; MAF, mutant allele fraction.
